# Induction of Germinal Centers by MMTV Encoded Superantigen on B Cells

**DOI:** 10.1155/2001/79823

**Published:** 2001

**Authors:** W. J. Simmons, M. Simms, R. Chiarle, F. Mackay, V. K. Tsiagbe, J. Browning, G. Inghirami, G. J. Thorbecke

**Affiliations:** 1 Department of Pathology and Comprehensive Kaplan Cancer Center New York University School of Medicine 550 First Avenue New York NY 10016 USA; 2 Biogen Corp. Cambridge MA 02142 USA

**Keywords:** germinal center(s), superantigen, Mtv7, follicular dendritic cells, lymphotoxin and tumor necrosis factor

## Abstract

It has not been established whether an endogenous superantigen (SAg) expressed on B cells can induce germinal centers (GCs). An interesting model is that of mammary tumor virus encoded viral SAgs, which induce vigorous T cell proliferation and are predominantly expressed on activated B cells. We have used this model to analyze the possibility that direct stimulation of Mtv7^+^ DBA/2 B cells by vSAg-responsive (Vβ6^+^) BALB/c T cells can give rise to GCs. Injection of BALB/c SCID mice iv with 2 × 10^6^ DBA/2 B cells, together with LPS, followed by 2 × 10^6^ BALB/c T cells induces numerous large splenic GCs within 3–5 days. The GCs are still large on day 7, but are very much reduced by day 10. B cell activation with LPS is needed for this effect. These GCs form in spite of the apparent absence of follicular dendritic cells (FDCs) as judged by staining for several FDC surface markers. Control mice receiving either BALB/c T or DBA/2 B cells + LPS alone or DBA/2 T + B cells + LPS fail to exhibit any GCs on days 3–7. Numerous small clusters of PNA^+^ cells, but few large GCs are observed when TNF-R(p55)-Ig is also injected, whereas LTβR-Ig treatment impeded the formation of aggregations of these cells even further, leaving scattered PNA+ single cells and very small clumps throughout the white pulp of the spleens. Anti-TNFα had no effect. These results suggest that endogenous vSAg mediated GC formation is independent of antigen trapping by FDCs.


*Developmental Immunology*, 2001, Vol. 8(3-4), pp. 201-211
Reprints available directly from the publisher
Photocopying permitted by license only

© 2001 OPA (Overseas Publishers Association)
N.V. Published by license under
the Harwood Academic Publishers imprint,
part of The Gordon and Breach Publishing Group,
a member of the Taylor and Francis Group

### Induction of Germinal Centers by MMTV Encoded Superantigen on B Cells*

W. J. SIMMONS[a], M. SIMMS[a], R. CHIARLE[a], F. MACKAY[b], V. K. TSIAGBE[a], J. BROWNING[b], G. INGHIRAMI[a] and G. J. THORBECKE[a†]

[a]*Department of Pathology and Comprehensive Kaplan Cancer Center, New York University School of Medicine, 550 First Avenue, New York, NY 10016 and* [b]*Biogen Corp., Cambridge, MA 02142*

It has not been established whether an endogenous superantigen (SAg) expressed on B cells can induce germinal centers (GCs). An interesting model is that of mammary tumor virus encoded viral SAgs, which induce vigorous T cell proliferation and are predominantly expressed on activated B cells. We have used this model to analyze the possibility that direct stimulation of Mtv7$^{+}$ DBA/2 B cells by vSAg-responsive (V$\beta$6$^{+}$) BALB/c T cells can give rise to GCs. Injection of BALB/c SCID mice iv with $2 \times 10^6$ DBA/2 B cells, together with LPS, followed by $2 \times 10^6$ BALB/c T cells induces numerous large splenic GCs within 3–5 days. The GCs are still large on day 7, but are very much reduced by day 10. B cell activation with LPS is needed for this effect. These GCs form in spite of the apparent absence of follicular dendritic cells (FDCs) as judged by staining for several FDC surface markers. Control mice receiving either BALB/c T or DBA/2 B cells + LPS alone or DBA/2 T + B cells + LPS fail to exhibit any GCs on days 3–7. Numerous small clusters of PNA$^{+}$ cells, but few large GCs are observed when TNF-R(p55)-Ig is also injected, whereas LT$\beta$R-Ig treatment impeded the formation of aggregations of these cells even further, leaving scattered PNA$^{+}$ single cells and very small clumps throughout the white pulp of the spleens. Anti-TNF$\alpha$ had no effect. These results suggest that endogenous vSAg mediated GC formation is independent of antigen trapping by FDCs.

*Keywords:* germinal center(s), superantigen, Mtv7, follicular dendritic cells, lymphotoxin and tumor necrosis factor

*Abbreviations:*FDC, follicular dendritic cells; GC, germinal center; LPS, endotoxin; MMTV, murine mammary tumor virus; PNA, peanut agglutinin; vSAg, viral superantigen

### INTRODUCTION

Germinal center (GC) formation during the immune response to exogenous traditional Ags is thought to require the interaction of B and T cells as well as follicular dendritic cells (FDCs) (Tsiagbe et al., 1996). Stimulation of a large number of T helper cells, such as occurs after injection of an exogenous superantigen (SAg), might cause the polyclonal activation of B cells in an antigen nonspecific fashion (Modlin et al.,

* This work was supported by USPHS Grant # AG-04980 from the National Institute of Aging.
† corresponding author. E-mail: thorbj01@mcrcr.med.nyu.edu

1996, Tumang et al., 1991, Luther et al., 1997), perhaps even leading to autoimmunity (Acha-Orbea, 1993, Ponzio et al., 1997). Bacterial and exogenous viral SAgs are also traditional antigens that can induce Ab formation and would therefore be expected to induce GC formation. However, the *endogenous* retroviral (v) SAgs, encoded by the LTR of mammary tumor viruses, are transcribed primarily in B and CD8 T cells (Webb and Sprent, 1990) and the protein is detectable on the surface of activated B cells and B cell lymphomas (Winslow et al., 1992, Mohan et al., 1993). Although vSAgs tend to bind strongly to MHC class II antigens (Winslow et al., 1994), and are probably not present as soluble products, they can move from class II$^-$ to class II$^+$ cells (Delcourt et al., 1997). They fail to induce detectable Ab responses and cytotoxic T cell responses (Matossian-Rogers and Festenstein, 1977, Herrmann et al., 1992), but induce strong proliferation of the CD4$^+$ T cells bearing the responsive V$\beta$ in their TCR, usually followed by deletion of these T cells, depending on the avidity of the interaction with the vSAg presenting cell (Webb et al., 1994).

In their study of the histological response to injection of exogenous MMTV, Luther et al (Luther, et al., 1997) concluded that vSAg induced GCs but with slower kinetics than traditional Ags. It was not clear from their data how the histological response to other viral Ags was distinguished from that to the vSAg. It has been shown that peanut agglutinin (PNA) binding, a typical property of GC B cells, may be upregulated on B cells during their interaction with allogeneic T cells (Forman and Pure, 1991). In view of the similarity in the magnitude of the proliferative response induced by vSAg and by allogeneic cells, we have therefore determined whether the *in vivo* response to endogenous vSAgs expressed on B cells is accompanied by GC formation and, if so, the kinetics of this T cell-induced B cell response in a SCID mouse environment where FDCs are missing (Kapasi et al., 1993). For these studies we have used the H2$^d$ strains, DBA/2 and BALB/c, which differ in expression of the strongly stimulating *Mtv7*-encoded vSAg (Mls1$^a$).

### MATERIALS AND METHODS

### Mice

Balb/c, Balb/c SCID and DBA2 mice were purchased from the National Cancer Institute (Bethesda, MD). Animals were maintained in pathogen free micro-isolator cages in the Animal Facility at NYU School of Medicine.

### Cell Isolations

B cells were negatively selected from donor spleen cell suspensions by treating with anti-T cell cocktail {containing mAb to CD4 (GK1.5), Thy1.2 (6.80), Lyt2.2 (HO-2.2) and Ly1.2 (C3PO)} for 30 min at 4°C, followed by lysis of the T cells with young rabbit C for 1h in Petri dishes at 37°C. Nonadherent B cells were washed 3x in Dulbecco's PBS prior to injection. T cells were negatively selected from donor lymph node cell suspensions by treating with anti-B cell cocktail {containing anti-IA$^d$ (MKD6), anti-B220 (RA3–3A1/6.1) and anti-CD24 (J11.D2)] and C as described above. Alternatively, total T cells or CD4 T cells were negatively selected by incubation of lymph node cells with rat anti-murine CD11b/Mac1 (M1/70.15; CALTAG, Burlingame, CA) and B220, with or without anti-CD8$\alpha$ (Pharmingen), followed by fractionation in a magnetic field after incubation with magnetic microbeads coated with anti-rat Ig from DYNAL (Lake Success, NY).

### Cell transfers

$2 \times 10^6$ B cells ± 5 $\mu$g lipopolysaccharide (LPS from E. Coli *0111:B4*; Difco Laboratories, Detroit, MI) were injected iv into Balb/c SCID recipients on day 0, followed by $2 \times 10^6$ T cells iv one day later. In some experiments, anti-TNF (TN3, kindly donated by Dr. R. D. Schreiber, Washington U. School of Medicine, St Louis, MO), TNF-R-Ig (p55) and LT$\beta$R-Ig, prepared as described (Browning et al., 1996), were injected (3–400 $\mu$g/mouse, ip) at the time of T cell transfer. Control cells or normal Ig preparations were injected as mentioned in results.

### Flow cytometry

$10^6$ cells in Hanks' BSS, 1% BSA and 0.1% sodium azide were incubated with 0.1 μg anti-CD4-PE, anti-CD8-TriColor, anti-Vβ6-FITC and/or anti-Vβ2-FITC (Pharmingen, San Diego, CA) and read in a FACScan (Beckton Dickenson, San Jose, CA).

### Immunohistochemistry

Tissues were fixed in Carnoy's fixative for 1h at RT and transferred to 60% ethanol until embedded in paraffin. In the case of PNA staining, endogenous peroxidase activity was quenched with 0.3% $H_2O_2$ in PBS (pH 7.2) for 5 min. Nonspecific activity was blocked by incubating the slides in 1% bovine albumin (Sigma, St. Louis, MO) for 15 min. Tissues were incubated with horseradish peroxidase (HRP) conjugated PNA (25 μg/ml, Sigma) for 1h, washed, developed with 0.08% diaminobenzidine (DAB) and 0.3 % $H_2O_2$, and counterstained with methyl green (Fluka, Ronkokoma, NY). Additional slides were stained with methyl green pyronin.

Spleens were also frozen at −77°C in OCT embedding medium and mounted for cryostat sectioning. Sections, 5–7 μm thick, were dried and fixed in acetone. After quenching of endogenous peroxidase activity, sections were incubated with mAbs (10 μg/ml) for 1 h at room temperature in a humidified chamber. HRP and alkaline phosphatase (AP) activities were developed using DAB and 5-bromo-4-chloro-3-indolyl phosphate/nitroblue tetrazolium (BCIP/NBT) from Sigma (St. Louis, MO), respectively. The stained sections were rinsed 5 min in methanol and counterstained with Giemsa. The rat antibodies used for staining were FDCM1 (4C11) and FDCM2 (209), both kindly donated by Dr. M. Vilbois-Kosco, anti-CR1 (8C12), anti-CD4 (RM4–5), and biotinylated anti-B220 (RA3–6B2), all purchased from Pharmingen. Developing reagents were goat anti-rat Ig-HRP and streptavidin-AP (Pharmingen).

### Mixed Lymphocyte Cultures

Nonadherent DBA/2 splenocytes were T cell depleted by treatment with an anti-T cell cocktail and C as described above. To prepare DBA/2 B cell blasts, B cells were cultured at $0.5 \times 10^6$ cells/ml + 50 μg LPS in Iscove's modified Dulbecco's medium (BioWhittaker, Walkersville, MD) supplemented with 10% fetal bovine serum (Gibco/BRL Grand Island, NY), 2mM L-glutamine and $5x10^{-5}$ M 2-mercaptoethanol for 48 h. Responder lymph node cells were prepared from BALB/c mice and placed in culture at $4 \times 10^5$ cells/well in 96 well plates with either $2 \times 10^4$ cells/well DBA/2 B cell blasts or resting B cells ± 20 μg/ml mAb to vSAg7 (Mls-1; Pharmingen) or control mouse Ig for 48 h. 1 μCi $^3$H-thymidine (Dupont-NEN, Boston, MA) was added to each well and 24 h later the cultures were harvested onto glass fiber filter. The amount of $^3$H incorporated was determined by liquid scintillation counting.

### Quantitation of Germinal Centers

GC areas were measured on PNA-stained slides using the Cell Analysis System Micrometer v.1 (Elmhurst, IL). Measurements were made on at least three traverse spleen sections per mouse and the percentage spleen area occupied by GCs was calculated.

### Statistical evaluation

Statistical significance was calculated using the Student t-test.

### RESULTS

### Germinal center formation as a result of T-B cell interaction during the vSAg-7 induced response

When SCID mice were injected with DBA/2 B cells and LPS, followed one day later by BALB/c T cells, GC formation could first be detected in the spleen on day 3 (Table I, Figs. 1 and 2). There was a rapid increase in both the size and number of GCs observed

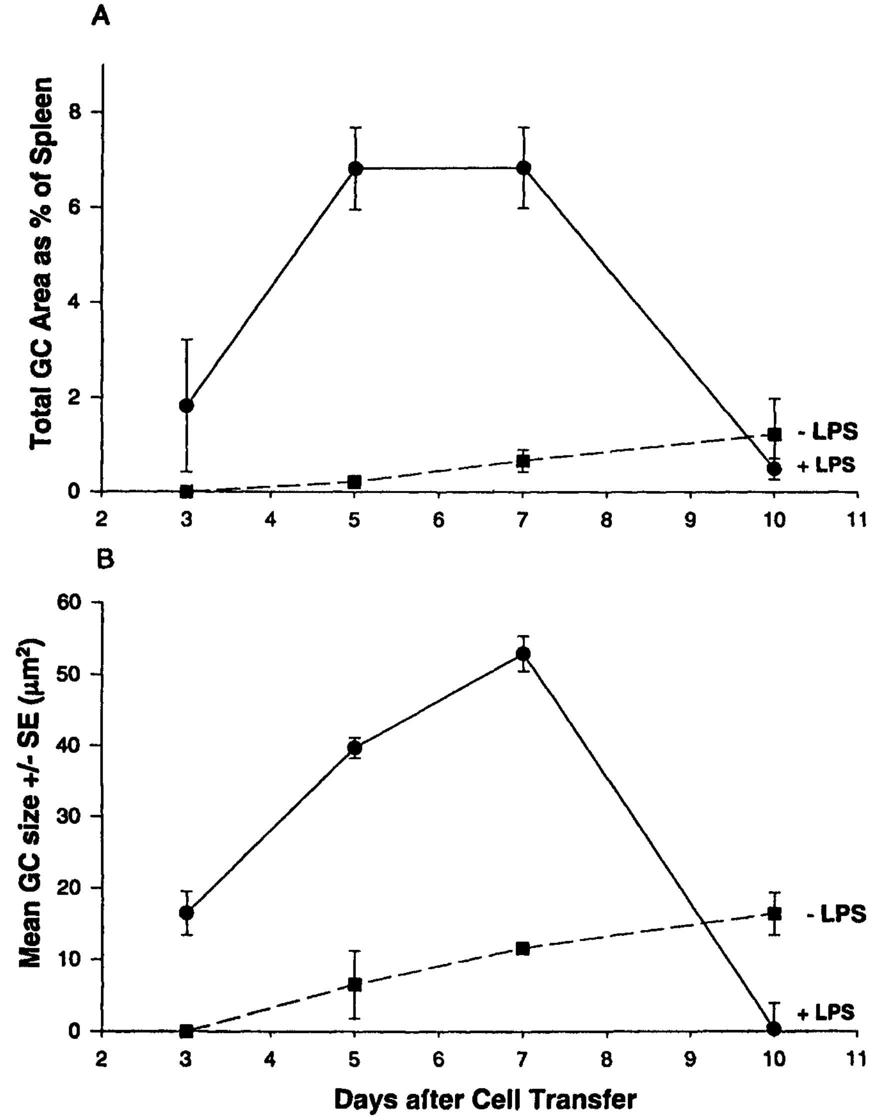

FIGURE 1 Germinal center development in SCID BALB/c mice after transfer of BALB/c T and DBA/2 B cells with and without LPS. Mice were injected iv with 2 x $10^6$ B cells with or without 5 µg E. coli endotoxin (LPS) on day 0. On day 1 they received 2 × $10^6$ T cells iv and their PNA-peroxidase stained spleen sections (at least three cross sections per spleen) were examined for the presence of GCs on the indicated days after B cell transfer. A: The measured GC areas in a recipient spleen were combined and expressed as a percentage of the spleen section areas examined for that mouse. Mean values ± SE are represented; the number of mice per group are given in Table I. B: The mean GC size ± SE for all the mice in the group are presented (n = number of mice in the group multiplied by the number of GC/mouse, as shown in Table I)

in the spleens of such recipients during the following days, such that at peak development more than 6% of the area of spleen sections was occupied by GCs. This was only seen when LPS was injected at the time of B cell transfer; without LPS, GC formation was minimal. However, the GC response was not induced by

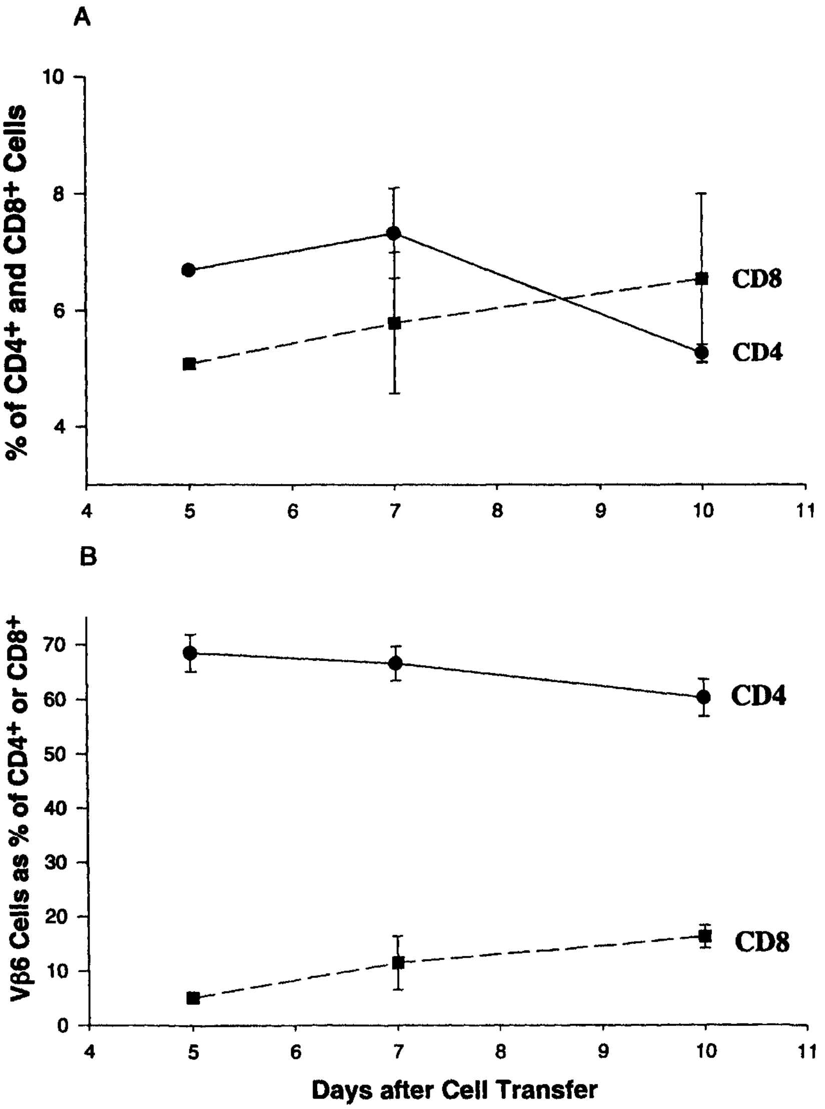

FIGURE 2 Vβ$6^+$ CD4 and CD8 T cell expansion in SCID BALB/c spleens after transfer of BALB/c T (on day 1) and DBA/2 B cells + LPS (on day 0). Spleen cell suspensions were prepared on the indicated days after B cell transfer and triple stained for the presence of Vβ$6^+$ cells in CD4 and CD8 T cells as described in materials and methods. Results are given of a single experiment and were representative of at least two other similar experiments. A: The representation of CD4 and CD8 cells in recipients' spleens (n = 2, 4, and 6 mice per group on days 5, 7, and 10, respectively) is given as mean percentages ± SE of total spleen cells. The ratio of CD4/CD8 in donor cells in this experiment was 2.2. Vβ$6^+$ cells as mean percentages ± SE of either CD4 or CD8 T cells in recipient spleens in the same experiment; Vβ$6^+$ cells comprised 10.6 and 7.4% of CD4 and CD8, respectively, in the donor cell preparation. Vβ2 cells were also measured, but remained below 1 % in recipients, although they comprised 7.5 and 6.9% of CD4 and CD8, respectively, in the donor cell preparation

LPS itself, because injection of LPS and DBA/2 B cells followed by DBA/2 T cells (or BALB/c B followed by BALB/c T cells) did not result in the appearance of GCs prior to day 10 (Table I). Thus, the rapid GC formation was dependent on the response of BALB/c T cells to LPS-activated DBA/2 B cells.

TABLE I Germinal Center Formation in SCID-BALB/c Recipients of BALB/c T and DBA/2 B cells

| *Cells Transferred*[a] | *Day After B cell transfer* | *GCs as % of Spleen Area Mean % ± SE ($n^b$)* | *GC Size*[c] *Mean $\mu^2$ ± SE* | *GC per Mouse*[d] |
|---|---|---|---|---|
| DBA/2 B + BALB/c T | 3 | 1.82 ± 1.4 (3) | 16.5 ± 3.1 | 7.7 |
| with LPS | 5 | 6.82 ± 0.86 (17) | 39.7 ± 1.5 | 11.9 |
| | 7 | 6.84 ± 0.85 (9) | 53.0 ± 2.4 | 10.4 |
| | 10 | 0.49 ± 0.22 (6) | 9.3 ± 3.6 | 1.5 |
| DBA/2 B + DBA/2 T | 3 | 0 (3) | | 0 |
| with LPS | 5 | 0.21 ± 0.13 (5) | 6.6 ± 4.7 | 1.2 |
| | 7 | 0.66 ± 0.24 (8) | 11.6 ± 2.5 | 2.8 |
| | 10 | 1.23 ± 0.76 (5) | 16.5 ± 3.0 | 4.0 |
| DBA/2 B + BALB/c T | 5 | 0.09 ± 0.09 (3) | 1.8 ± 1.8 | 1.0 |
| without LPS | 7 | 0.33 ± 0.33 (3) | 6.7 ± 6.7 | 1.0 |

a. B cells +/- 5 μg LPS were injected iv on day 0 and T cells on day 1. No GCs were observed in recipients of DBA/2 B or BALB/c T cells alone.
b. n = Number of mice in group.
c. The mean sizes ± SE for all GCs measured in the mice of that group.
d. Number of germinal centers observed in 3 spleen sections per mouse..

In most experiments in which total BALB/c T cells were used, the acute GC response had disappeared by day 10 (Figs. 1A and B). In fact, by day 10 there was no longer any difference between mice receiving DBA/2 or BALB/c T cells, or between mice receiving LPS or no LPS at the time of B cell transfer. Those GCs that were still visible on day 10 were much smaller, and contained far less mitotic activity and apoptotic cells than the GCs observed earlier. However, in experiments with purified CD4 T cells and in one experiment with highly purified total T cells, in which the CD8 T cells failed to expand after transfer, see below, the large GCs were still present on day 10.

### Nature of BALB/c T cell response to DBA/2 LPS-induced blasts

Since it has been shown that the response to vSAg7 is dominated by $V\beta6^+$ T cells (Waanders and McDonald, 1992, Hayden et al., 1996), a comparison was made between the frequency of $V\beta6^+$ and $V\beta2^+$ T cells in the recipient spleens. At the time of cell transfer, the donor cells contained 9.5–11.5% $V\beta6^+$ and 6.8–7.6% $V\beta2^+$ cells in the $CD4^+$ and 7.4–12.4% $V\beta6^+$ and 5.5–6.9% $V\beta2^+$ cells in the $CD8^+$ population. $V\beta2^+$ T cells remained barely detectable in recipient spleens throughout the experiment, but $V\beta6^+$ cells expanded to above 60% of the total $CD4^+$ T cells in the spleen (Fig. 2B) within 4 days and remained this high through day 9 after T cell transfer. $CD8^+$ T cells increased slowly and less impressively, but in some experiments reached 20% of $CD8^+$ T cells by day 9 (Fig. 2B). The increase in Vβ6 in T cells was absent when LPS had not been injected at the time of B cell transfer, remaining below 2%. The ratio of $CD4^+/CD8^+$ T cells in the recipient spleens remained stable on days 4 and 6 after T cell transfer. In some experiments there was an increase in $CD8^+$ T cells by day 9 (Fig. 2A), suggesting that a cytotoxic T cell response might be taking place against the DBA/2 cells, which was not, however, dominated by $V\beta6^+$ cells to the same extent as was the response in the $CD4^+$ T cells. In experiments with purified donor CD4 T cells and also in some experiments with purified total T cells, this increase in CD8 T cells was not observed and the CD4/CD8 ratio in recipients' spleens by day 9 was approximately 10. In those experiments, GC formation in recipient spleens remained at peak levels through day 9 after T cell (or day 10 after B cell) transfer.

TABLE II Mixed Lymphocyte Response in BALB/c T cells is Mainly due to vSAg7

| *Stimulator Used* | *Reagents Added* | *Δ cpm ± SE*[a] | *% of Control Response* |
|---|---|---|---|
| γ-DBA/2 – LPS blasts | Control Ig | 8, 738 ± 2,100 | |
| | Anti-vSAg7 | 2, 083 ± 221 | 23.8 |
| γ-DBA/2 B cells | Control Ig | 1, 632 ± 86 | |
| | Anti-vSAg7 | 883 ± 55 | 54.2 |
| Con A | Control Ig | 6, 338 ± 265 | |
| | Anti-vSAg7 | 10, 877 ± 639 | 166.2 |

a. Means ± SE of 5 replicate cultures.

The *in vitro* proliferative response of BALB/c T cells to LPS-induced DBA/2 B cell blasts was studied for its sensitivity to inhibition by anti-vSAg7 mAb. The results in Table II show that the response to γ-irradiated LPS-blasts was much greater than that to unstimulated DBA/2 B cells. In addition, this response was ~80% inhibited by the presence of anti-vSAg7. This inhibition was specific as shown by the lack of inhibition of the response to Con A. These data indicate that the initial response of BALB/c T cells to DBA/2 LPS blasts is overwhelmingly due to vSAg7.

**Role of molecules of the TNF-R family in the vSAg7-induced GC response**

The rapidity of the GC formation after B + T cell transfer in the SCID mouse microenvironment suggested a relative independence from FDCs, which have been reported to be undetectable in SCID lymphoid tissues (Kapasi, et al., 1993). In fact, staining for CR1, FDC-M1 and FDC-M2 failed to detect any FDCs on day 5 after injection of DBA/2 B + BALB/c T cells (not shown), although the spleen contained numerous GCs. To study this aspect further, and in view of the well-known inhibitory effect of TNF-RI-Ig and LTβR-Ig on FDC development (Matsumoto et al., 1996, Rennert et al., 1997), we injected 0.4 mg TNF-RI(p55)-Ig or LTβR-Ig ip at the time of the B cell-transfer. Although this resulted in a much more disperse response on the part of the $PNA^+$ B cells, probably as a result of the known destructive influence of these reagents on the B-T cell compartments and follicular structure, the total number of PNA-binding blast cells seen in small clusters all over the white pulp of the spleen was still measurable (Figs. 3 and 4). The number of GCs per spleen was higher in the TNF-RI-Ig injected than in control-Ig injected recipients on day 5 after B cell transfer, while the mean GC size was much reduced (Fig. 3). The effect of LTβR-Ig was more pronounced than that of TNF-R-Ig and resulted in the presence of very small clumps of PNA binding cell clusters all over the periarteriolar sheaths ("white pulp") of the spleen without any recognizable real GCs on day 5. By day 7 there was some recovery from this effect and GCs could now be recognized. No effect was observed from the injection of 0.25 mg anti-TNF-α, injected together with the B cells, on GC formation.

### DISCUSSION

These results show that LPS activated vSAg7 expressing B cells rapidly form GCs when stimulated by T cells responding to the vSAg. The GC formation becomes detectable as early as three days after the B or two days after the T cell transfer, which is at least 24–48 h sooner than after a primary injection of nominal Ag into a normal animal (Tsiagbe, et al., 1996, Langevoort et al., 1963, Jacobson and Thorbecke, 1968, Coico et al., 1983). The vSAg induced GC response resembles more that of a secondary response to nominal Ag, where the presence of primed T cells causes an acceleration in GC formation (Buerki et al., 1989). This rapidity is undoubtedly the result of the

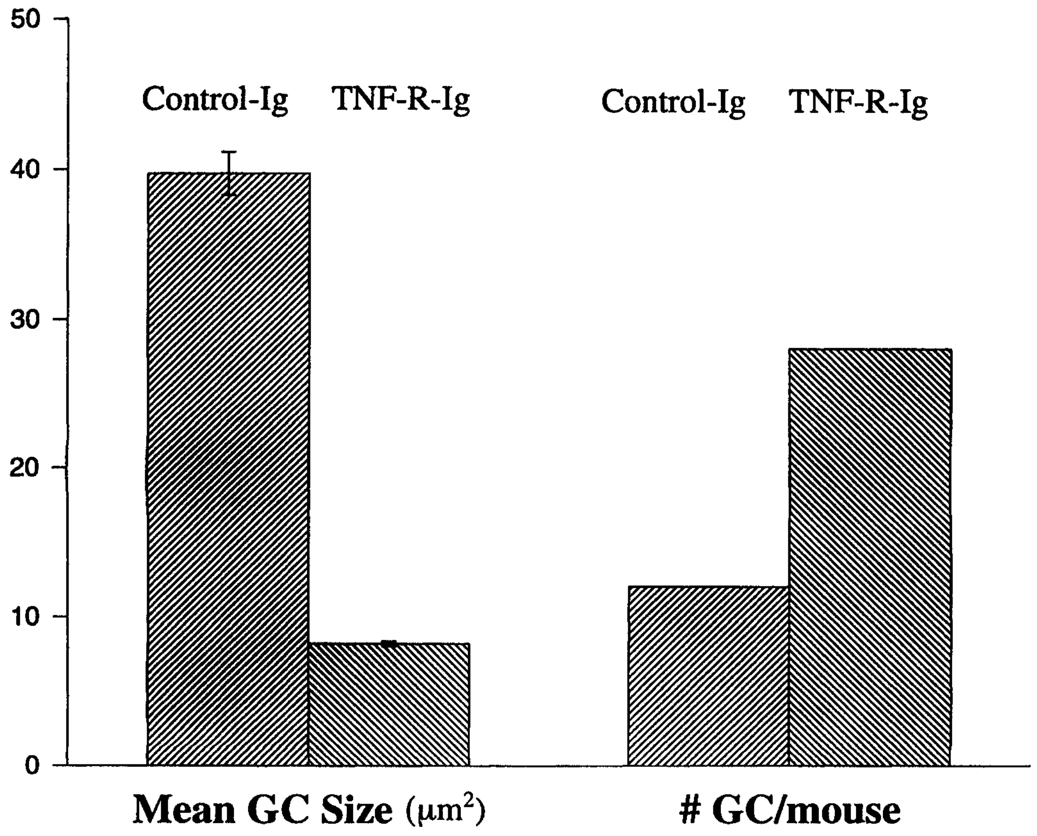

FIGURE 3 Effect of TNF-R1-Ig on germinal center formation in SCID BALB/c spleens after transfer of BALB/c T (on day 1) and DBA/2 B cells + LPS (on day 0). Mice received cell transfers as in Fig. 1. One group of 4 mice was also injected on day 0 with 0.4 mg TNF-R1-Ig ip, the other group received control Ig. The mice were killed on day 5. The results are expressed as mean GC size ± SE for each group of mice (left) and as the numbers of GCs counted in 3 spleen sections per mouse vigorous Vβ6 CD4 T cell response that is induced by the LPS activated, but not resting DBA/2 B cells.

The response is not only very rapid, but also shorter lived than the GC response to a nominal Ag given without adjuvants, which usually starts to wane only after day 14, and is detectable in the spleen for approximately three weeks (Langevoort, et al., 1963, Thorbecke et al., 1962). The acute disappearance of GCs between days 7 and 10 in the present experiments could have two major explanations. In the first place, although vSAg7 itself is known not to induce a cytotoxic response (Herrmann, et al., 1992), there might have been a cytotoxic T cells response by the BALB/c CD8 T cells against other minor histocompatibility antigens on the DBA/2 cells. CD8 T cells predominated in the recipient spleens by day 10 and could be responding against such Ags on the GC B cells of DBA/2 origin. The results obtained with the purified CD4 T cell transfers, where GCs remained large in recipients at least through day 10, suggest that this explanation may be correct. However, future experiments in which congenic BALB/c mice which express vSAg7 are used as the B cell donors will determine whether such a cytotoxic response causes the disappearance of the GCs in these experiments.

A second reason for the GC disappearance after day 7, could be a lack of interaction with FDCs, either because of a relative absence of FDCs, or because of

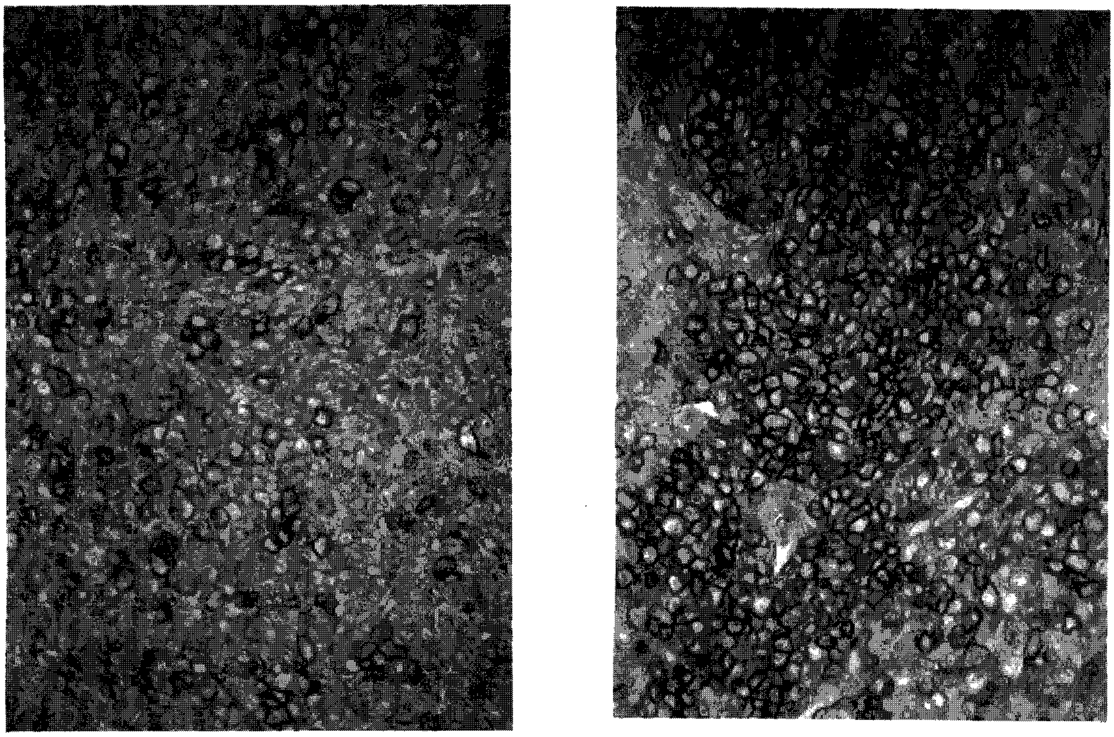

FIGURE 4 Germinal center appearance in spleens from TNF-R1-Ig treated recipients (left) as compared to controls (right) on day 5 after B cell transfer. Spleen sections were stained with PNA-peroxidase and methylgreen. Magnification 100x the lack of interaction of the proliferating B cells in these vSAg induced GCs with immune complexes on the surface of FDCs, resulting in enhanced apoptosis of the GC B cells. It has been shown conclusively that the interaction between GC B cells and FDCs is important for the survival of the B cells (Koopman et al., 1994) and that immune complexes with bound complement present on the FDC surface greatly contribute to this effect (Carroll and Fischer, 1997, Croix et al., 1996). Indeed, staining for FDCs in the SCID recipients in our experiments showed a complete absence of detectable FDCs on day 5 when numerous large GCs were present in the follicles. These results agree with those obtained in LT$\beta^{-/-}$ mice, where FDCs are not found, but GCs are produced in the mesenteric lymph nodes that sometimes develop in these mice (Fu et al., 1997, Koni et al., 1997, Koni and Flavell, 1998). These GCs are localized within $CD24^{+}IgD^{+}$ B cell containing follicles, are specifically induced by antigen challenge ip, but fail to persist (Koni and Flavell, 1999). Thus, both the normal localization of the GCs in follicles and a failure to persist are shown in two different situations, where functional interaction between GC B cells and FDCs is lacking. The effect of the injection of TNF-R-Ig on GC formation in the present experiments provides further evidence that the production of clusters of PNA-binding B cells does not require participation from FDCs. The disorganization of the lymphoid tissue, which is reported to result from the injection of either TNF-R-Ig or LTβR-Ig (Matsumoto, et al., 1996, Rennert et al., 1996, Matsumoto et al., 1997), evidently prevents the normal localization of the GC response in follicles, although most of the clusters of PNA-binding blast cells observed are still predominantly localized in the white rather than in the red pulp of the recipient mouse spleens.

B cell lymphomas of SJL mice require CD4 T cells for their growth and development. These lymphomas start in germinal centers (GCs) and express an *Mtv29*-encoded vSAg which strongly stimulates syngeneic V$\beta$16$^+$CD4 T cells. Thus, the observation in the present study that, in the presence of vSAg responsive T cells, vSAg expressed on B cells can induce GC formation, strongly supports the suggestion that vSAg expression on normal GC cells in SJL mice may cause abnormally vigorous GC production, possibly creating a condition permissive for lymphoma formation (Tsiagbe et al., 1998, Ponzio et al., 1996).

### Acknowledgements

The technical assistance of Larry Richards in the preparation of histological sections is gratefully acknowledged. We also greatly appreciate the photographic expertise of Thomas Semkow.

### References

Acha-Orbea H. (1993). Bacterial and viral superantigens: roles in autoimmunity? Ann Rheum Dis **52 Suppl 1**:S6–16.

Browning J.L., Miatkowski K., Griffiths D.A., Bourdon P.R., Hession C., Ambrose C.M. and Meier W. (1996). Preparation and characterization of soluble recombinant heterotrimeric complexes of human lymphotoxins alpha and beta. J Biol Chem **271**:8618–8626.

Buerki H., Kraft R., Hess M.W., Laissue J., Cottier H. and Stoner R.D. (1989). Germinal center kinetics in lymph nodes of primed mice stimulated with complexed as opposed to free antigen. Immunol Lett **23**:87–94.

Carroll M.C. and Fischer M.B. (1997). Complement and the immune response. Curr Opin Immunol **9**:64–69.

Coico R.F., Bhogal B.S. and Thorbecke G.J. (1983). Relationship of germinal centers in lymphoid tissue to immunologic memory. VI. Transfer of B cell memory with lymph node cells fractionated according to their receptors for peanut agglutinin. J Immunol **131**:2254–2257.

Croix D.A., Ahearn J.M., Rosengard A.M., Han S., Kelsoe G., Ma M. and Carroll M.C. (1996). Antibody response to a T-dependent antigen requires B cell expression of complement receptors. J Exp Med **183**:1857–1864.

Delcourt M., Thibodeau J., Denis F. and Sekaly R.P. (1997). Paracrine transfer of mouse mammary tumor virus superantigen. J Exp Med **185**:471–480.

Forman M.S. and Pure E. (1991). T-independent and T-dependent B lymphoblasts: helper T cells prime for interleukin 2-induced growth and secretion of immunoglobulins that utilize downstream heavy chains. J Exp Med **173**:687–697.

Fu Y.X., Huang G., Matsumoto M., Molina H. and Chaplin D.D. (1997). Independent signals regulate development of primary and secondary follicle structure in spleen and mesenteric lymph node. Proc Natl Acad Sci U S A **94**:5739–5743.

Hayden K.A., Tough D.F. and Webb S.R. (1996). In vivo response of mature T cells to Mlsa antigens. Long-term progeny of dividing cells include cells with a naive phenotype. J Immunol **156**:48–55.

Herrmann T., Waanders G.A., Chvatchko Y. and MacDonald H.R. (1992). The viral superantigen Mls-1a induces interferon-gamma secretion by specifically primed CD8+ cells but fails to trigger cytotoxicity. Eur J Immunol **22**:2789–2793.

Jacobson E.B. and Thorbecke G.J. (1968). Relationship of germinal centers in lymphoid tissue to immunologic memory. IV. Formation of 19S and 7S antibody by splenic white and red pulp during the secondary response in vitro. Lab Invest **19**:635–642.

Kapasi Z.F., Burton G.F., Shultz L.D., Tew J.G. and Szakal A.K. (1993). Induction of functional follicular dendritic cell development in severe combined immunodeficiency mice. Influence of B and T cells. J Immunol **150**:2648–2658.

Koni P.A. and Flavell R.A. (1998). A role for tumor necrosis factor receptor type 1 in gut-associated lymphoid tissue development: genetic evidence of synergism with lymphotoxin beta. J Exp Med **187**:1977–1983,.

Koni P.A. and Flavell R.A. (1999). Lymph node germinal centers form in the absence of follicular dendritic cell networks. J Exp Med **189**:855–864.

Koni P.A., Sacca R., Lawton P., Browning J.L., Ruddle N.H. and Flavell R.A. (1997). Distinct roles in lymphoid organogenesis for lymphotoxins alpha and beta revealed in lymphotoxin beta-deficient mice. Immunity **6**:491–500.

Koopman G., Reutelingsperger C.P., Kuijten G.A., Keehnen R.M., Pals S.T. and van Oers M.H. (1994). Annexin V for flow cytometric detection of phosphatidylserine expression on B cells undergoing apoptosis. Blood **84**:1415–1420.

Langevoort H.L., Asofsky R.M., Jacobson E.B., deVries T. and Thorbecke G.J. (1963). Gamma globulin and antibody formation *in vitro* II. Parallel observations on histologic changes and antibody formation in the white and red pulp of the rabbit spleen during the primary response with special reference to the effect of endotoxin. J. Immunol. **90**:60.

Luther S.A., Gulbranson-Judge A., Acha-Orbea H. and MacLennan I.C. (1997). Viral superantigen drives extrafollicular and follicular B cell differentiation leading to virus-specific antibody production. J Exp Med **185**:551–562.

Matossian-Rogers A. and Festenstein H. (1977). Cytostatic effector cells generated in vivo against M locus determinants. Clin Exp Immunol **27**:335–339.

Matsumoto M., Fu Y.X., Molina H. and Chaplin D.D. (1997). Lymphotoxin-alpha-deficient and TNF receptor-I-deficient mice define developmental and functional characteristics of germinal centers. Immunol Rev **156**:137–144.

Matsumoto M., Mariathasan S., Nahm M.H., Baranyay F., Peschon J.J. and Chaplin D.D. (1996). Role of lymphotoxin and the type I TNF receptor in the formation of germinal centers. Science **271**:1289–1291.

Modlin C.S., Muruve N.A., Stanko D., Caulfield M.J. and Fairchild R.L. (1996). Recipient polyclonal B cell activation and immunoglobulin production induced by priming with a retroviral superantigen. Cell Immunol **169**:252–263.

Mohan N., Mottershead D., Subramanyam M., Beutner U. and Huber B.T. (1993). Production and characterization of an Mls-1-specific monoclonal antibody. J Exp Med **177**:351–358.

Ponzio N.M., Tsiagbe V.K. and Thorbecke G.J. (1996). Superantigens related to B cell hyperplasia. Springer Semin Immunopathol **17**:285–306.

Ponzio N.M., Zhang D.J., Tsiagbe V.K. and Thorbecke G.J. (1997). Influence of MTV superantigen on B cell lymphoma development in SJL/J mice. In Viral Superantigens, pp. 219–231. CRC Press, Boca Raton, FL.

Rennert P.D., Browning J.L. and Hochman P.S. (1997). Selective disruption of lymphotoxin ligands reveals a novel set of mucosal lymph nodes and unique effects on lymph node cellular organization. Int Immunol **9**:1627–1639.

Rennert P.D., Browning J.L., Mebius R., Mackay F. and Hochman P.S. (1996). Surface lymphotoxin alpha/beta complex is required for the development of peripheral lymphoid organs. J Exp Med **184**:1999–2006.

Thorbecke G.J., Asofsky R.M., Hochwald G.M. and Siskind G.W. (1962). Gamma globulin and antibody formation in *vitro*. III. Induction of secondary response at different intervals after the primary: the role of secondary nodules in the preparation for the secondary response. J. Exp. Med. **116**:295–309.

Tsiagbe V.K., Inghirami G. and Thorbecke G.J. (1996). The physiology of germinal centers. Crit. Rev. Immunol. **16**:381–421.

Tsiagbe V.K., Ponzio N.M., Erianne G.S., Zhang D.J., Thorbecke G.J. and Inghirami G. (1998). Germinal center derived lymphomas in humans and mice. In The biology of germinal centers in lymphoid tissue (G. J. Thorbecke and V. K. Tsiagbe, eds.), pp. 199–234. Springer-Verlag, New York.

Tumang J.R., Cherniack E.P., Gietl D.M., Cole B.C., Russo C., Crow M.K. and Friedman S.M. (1991). T helper cell-dependent, microbial superantigen-induced murine B cell activation: polyclonal and antigen-specific antibody responses. J Immunol **147**:432–438.

Waanders G.A. and McDonald H.R. (1992). Hierarchy of responsiveness in vivo and in vitro among T cells expressing distinct Mls-1a-reactive v beta domains. Eur J Immunol **22**:291–293.

Webb S.R., Hutchinson J., Hayden K. and Sprent J. (1994). Expansion/deletion of mature T cells exposed to endogenous superantigens in vivo. J Immunol **152**:586–597.

Webb S.R. and Sprent J. (1990). Induction of neonatal tolerance to Mlsa antigens by CD8+ T cells. Science **248**:1643–1646.

Winslow G.M., Marrack P. and Kappler J.W. (1994). Processing and major histocompatibility complex binding of the MTV7 superantigen. Immunity **1**:23–33.

Winslow G.M., Scherer M.T., Kappler J.W. and Marrack P. (1992). Detection and biochemical characterization of the mouse mammary tumor virus 7 superantigen (Mls-1a). Cell **71**:719–730.

